# Longitudinal changes in skeletal muscle mass in patients with advanced squamous cell lung cancer

**DOI:** 10.1111/1759-7714.13958

**Published:** 2021-04-07

**Authors:** Jongsoo Lee, Eun Young Kim, Eunji Kim, Kwang Gi Kim, Young Jae Kim, Young Saing Kim, Hee Kyung Ahn, Sang‐Woong Lee

**Affiliations:** ^1^ Department of Radiology Gil Medical Center, Gachon University College of Medicine Incheon Republic of Korea; ^2^ Department of Biomedical Engineering Gachon University College of Medicine Incheon Republic of Korea; ^3^ Department of Internal Medicine Gil Medical Center, Gachon University College of Medicine Incheon Republic of Korea; ^4^ Division of Software School of AI‐SW, Gachon University Seongnam Republic of Korea

**Keywords:** cancer cachexia, chemotherapy, lung cancer, prognosis, sarcopenia

## Abstract

**Background:**

Skeletal muscle depletion (sarcopenia) is associated with poor prognosis in patients with lung cancer. We analyzed changes in skeletal muscle area using serial computed tomography (CT) until the death of patients with advanced squamous cell lung cancer (SQCLC).

**Methods:**

This retrospective study comprised 70 consecutive patients who underwent palliative chemotherapy for SQCLC. The cross‐sectional area of the skeletal muscle at the level of the first lumbar vertebra (L1) was measured using chest CT. An artificial intelligence algorithm was developed and used for the serial assessment of the muscle area. Sarcopenia was defined as an L1 skeletal muscle index <46 cm^2^/m^2^ in men and < 29 cm^2^/m^2^ in women.

**Results:**

The median age was 69 years; 62 patients (89%) had metastatic disease at the time of initial diagnosis. Sarcopenia was present in 58 patients (82.9%) at baseline; all patients experienced net muscle loss over the disease trajectory. The median overall survival was 8.7 (95% confidence interval 5.9–11.5) months. The mean percentage loss of skeletal muscle between the first and last CT was 16.5 ± 11.0%. Skeletal muscle loss accelerated over time and was the highest in the last 3 months of life (*p* < 0.001). Patients losing skeletal muscle rapidly (upper tertile, >3.24 cm^2^/month) had shorter overall survival than patients losing skeletal muscle slowly (median, 5.7 vs. 12.0 months, *p* < 0.001).

**Conclusions:**

Patients with advanced SQCLC lose a significant amount of skeletal muscle until death. The rate of muscle area reduction is faster at the end of life.

## Abstract 2

Patients with advanced SQCLC lose a significant amount of skeletal muscle until death. The rate of muscle area reduction is faster at the end of life.

## INTRODUCTION

Cancer cachexia is a multifactorial syndrome characterized by an ongoing loss of skeletal muscle mass (SMM) that cannot be fully recovered by conventional nutritional support, leading to progressive impairment of body functions.^1^ It is a common feature in advanced cancer and the leading cause of death in 20% of cancer patients.^2^ It is characterized by anorexia, reduced appetite, metabolic changes, and weight loss.^3^ As a key component of cancer cachexia, sarcopenia (skeletal muscle depletion) is highly prevalent and is associated with functional impairment, increased risk of chemotherapy‐related toxicities, and poor prognosis in patients with various cancers, including lung cancer.^4^, ^5^, ^6^, ^7^, ^8^, ^9^ However, the risk of sarcopenia can vary according to age, baseline constitution, tumor type, disease stage, treatment, and chemotherapy regimen.^10^ The evaluation of longitudinal changes in muscle wasting would be helpful in understanding the disease course and preparing for the end of life in advanced‐stage cancer patients.

Lung cancer is the leading cause of cancer‐related deaths worldwide.^11^ Non‐small‐cell lung cancer (NSCLC), which comprises mainly adenocarcinoma and squamous cell lung carcinoma (SQCLC), accounts for 80–85% of all lung cancer cases.^12^ SQCLC represents approximately 30% of all cases of NSCLC.^13^ Among patients with NSCLC, cancer cachexia and sarcopenia commonly occur and they are strongly associated with poor prognosis.^14^ However, the studies focused on the presence of sarcopenia at the time of diagnosis of lung cancer. Thus, the primary objective of this study was to analyze the longitudinal changes in skeletal muscle area with follow‐up computed tomography (CT) examinations from the time of diagnosis until the end of life in patients with advanced SQCLC.

## PATIENTS AND METHODS

### Patients

This retrospective study comprised 70 consecutive patients with advanced SQCLC who underwent palliative chemotherapy at Gachon University Gil Medical Center (GUGMC, Incheon, Korea) between September 2010 and March 2015. The medical records of all patients were reviewed, and they included age, sex, performance status (PS), smoking status, tumor‐node‐metastases stage, chemotherapy regimen, tumor response, and survival status. PS was estimated according to the Eastern Cooperative Oncology Group system. Tumor responses were classified using the Response Evaluation Criteria in Solid Tumors (RECIST) version 1.1. The institutional review board of our hospital approved this retrospective study and waived the requirement for informed patient consent; patient confidentiality was maintained throughout the study (approval number: GAIRB 2020‐447).

### Image analysis

Chest CT was performed using multidetector helical CT scanners (SOMATOM Definition Flash, Siemens Healthcare) with the following acquisition parameters: 1‐mm collimation, 120–140 kV, 75–350 mA, 0.75–1 s scan time, and 1–2 mm slice thickness during full inspiration.

Single cross‐sectional areas of the total skeletal muscle at the level of the first lumbar vertebra (L1) were measured using chest CT. In principle, a single cross‐sectional area of the total skeletal muscle at the third lumbar vertebra level (L3 muscle area, L3MA) is the most representative measure for estimating total body SMM and the L3 muscle index (L3MI, L3MA/height^2^) is used to determine sarcopenia.^15^, ^16^ However, the L1 muscle index (L1MI) served as the reference standard in our study because chest CT usually do not extend to the L3 level. According to our previous study, L1MI was highly associated with L3MI, and based on the relationship between L1MI and L3MI, the corresponding cutoff value of L1MI for determining sarcopenia was <46 cm^2^/m^2^ in men and 29 cm^2^/m^2^ in women.^17^

Skeletal muscle area was quantified using an in‐house software (Gachon_DeepBody developed in GUGMC, Incheon, Korea). For the Gachon_DeepBody software, U‐Net architecture was used.^18^ For hyper‐parameters, the batch size was set at 8, the learning rate was 0.0001, the optimizer algorithm was Adam, and the number of epochs was 150. The Gachon_DeepBody software isolates the muscle, subcutaneous fat, and visceral fat areas using the trained deep learning model and measures the area for each body composition. Using the software, skeletal muscles were automatically identified and the area was calculated (Hounsfield units [HU]: from −29 to 150 for skeletal muscle) using CT.

### Statistical analysis

Descriptive statistics are reported as proportions or means. Welch's ANOVA with Dunnett's T3 method was used to compare muscle loss rates between different periods for multiple comparisons. The time from the first CT until death was defined as overall survival (OS). The Kaplan–Meier method and log‐rank test were used to calculate and compare OS. Multivariable Cox regression analysis was performed to identify significant prognostic factors of OS. Variables with *p* values of <0.05 by univariable analysis were included in the multivariable model, which was adjusted for age, stage, and PS. In all assessments, statistical significance was accepted at the 95% confidence interval (*p* < 0.05). All statistical analyses were performed using SPSS Statistics for Windows ver. 25.0 (SPSS Inc.).

## RESULTS

### Characteristics of the study population

The baseline characteristics of the 70 study subjects are summarized in Table 1. The median patient age was 69 years and 62 patients (88.6%) were men. Among the 70 patients, 62 (88.6%) had metastatic disease and sarcopenia was present in 58 (82.9%) at baseline.

**TABLE 1:** Patient demographics (*n* = 70)

Characteristic	No. of patients	%
Age (years)		
Median (range)	69 (50–84)	
≥ 70	34	48.6
Male sex	62	88.6
ECOG performance status		
0–1	47	67.1
2	23	32.9
Smoking status		
Current	37	52.9
Ex‐smoker	25	35.7
Never‐smoker	8	11.4
Stage at treatment		
IIIB	8	11.4
IV	62	88.6
First‐line chemotherapy		
Combination therapy	59	84.3
Single‐agent	11	15.7
Response to first‐line chemotherapy		
Yes	27	38.6
No	43	61.4
First‐line chemotherapy regimen		
Gemcitabine/platinum	48	68.6
Paclitaxel/platinum	5	7.1
Gemcitabine/paclitaxel	6	8.6
Gemcitabine	9	12.9
Paclitaxel	2	2.9
Receipt of second‐line therapy	46	65.7
Second‐line regimen		
Docetaxel	21	30.0
EGFR TKIs^a^	18	25.8
Platinum‐based combination^b^	4	5.7
Vinorelbine	2	2.9
Pemetrexed	1	1.4
Sarcopenia	58	82.9

^a^ Includes erlotinib (*n* = 11), gefitinib (*n* = 5), and afatinib (*n* = 2).

^b^ Includes docetaxel/cisplatin (*n* = 2), vinorelbine/cisplatin (*n* = 1), and irinotecan/carboplatin (*n* = 1).

*Abbreviations*: ECOG, Eastern Cooperative Oncology Group; EGFR TKIs, epidermal growth factor receptor tyrosine kinase inhibitors.

### Median overall survival

**FIGURE 1:**
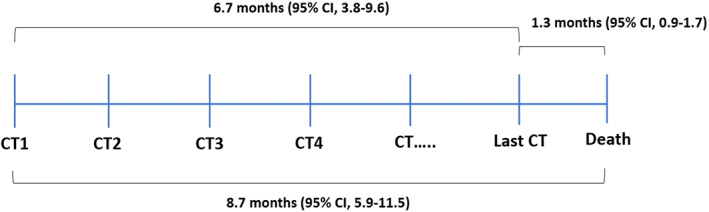
Timeline of the disease trajectory

The median OS for all patients with advanced SQCLC was 8.7 (95% confidence interval [CI] 5.9–11.5) months. The median interval between the last CT and deaths was 1.3 (95% CI 0.9–1.7) months. All patients experienced net muscle loss over the disease trajectory (Figure 1).

### Skeletal muscle area on follow‐up CT

**FIGURE 2:**
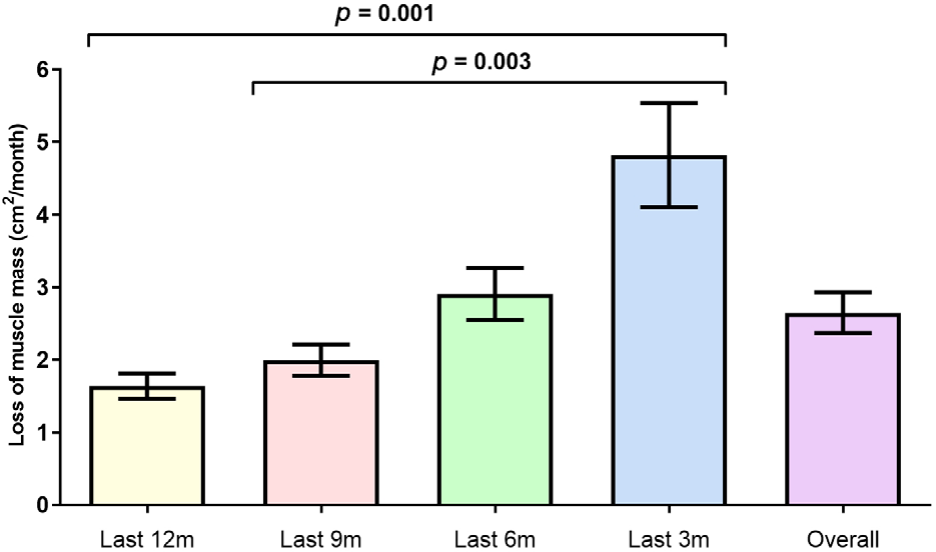
Rate of skeletal muscle loss. Skeletal muscle loss accelerated over time and was highest during the last 3 months

The mean percentage loss of skeletal muscle area between the first and last CT was 16.5 ± 11.0% (range 0.6–41.8%) and the mean decrease in muscle area was 17.28 ± 13.00 cm^2^ at a rate of 2.64 ± 2.36 cm^2^/month. Skeletal muscle loss accelerated over time and was the highest during the last 3 months (*p* < 0.001) (Table 2 and Figure 2).

**TABLE 2:** Skeletal muscle change

Period	Rate of skeletal muscle loss (cm^2^/month)	*p* value
	Mean ± SD	
Overall	2.64 ± 2.36	<0.001
During the last 12 months	1.64 ± 1.02	
During the last 9 months	2.00 ± 1.52	
During the last 6 months	2.91 ± 2.69	
During the last 3 months	4.82 ± 4.59	

*Abbreviation*: SD, standard deviation.

**FIGURE 3:**
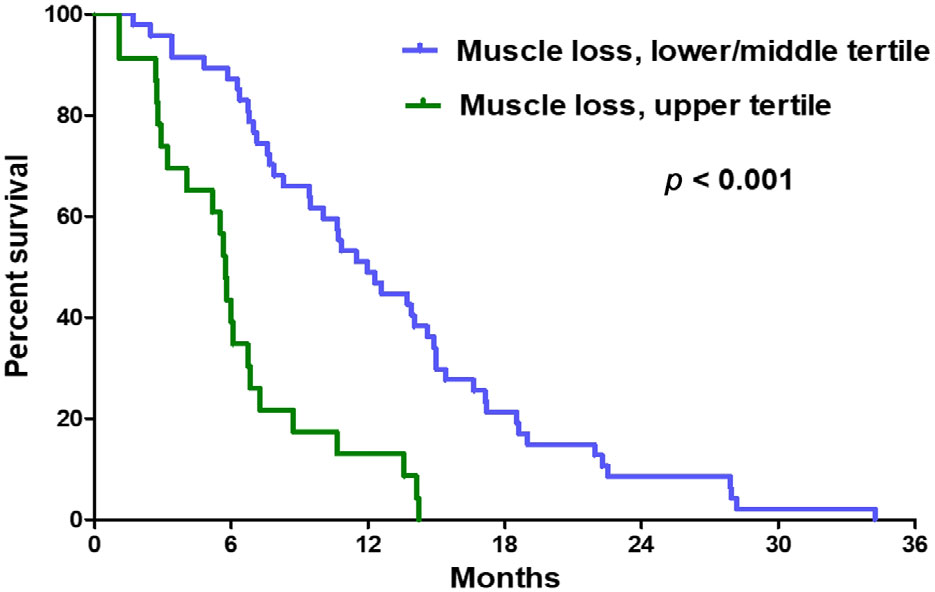
Survival curves according to the rate of skeletal muscle loss. Patients who had rapid skeletal musle loss (change of skeletal muscle area at ≥3.24 cm^2^/month, upper tertile) showed a shorter overall survival than patients who lost skeletal musle area at a slower rate (median 5.7 vs. 12.0 months, *p* < 0.001)

Furthermore, patients who lost skeletal musle area at ≥3.24 cm^2^/month (upper tertile) had a shorter OS than patients who lost skeletal musle area at a slower rate (median 5.7 vs. 12.0 months, *p* < 0.001) (Figure 3). The results of univariable and multivariable analyses of prognostic factors for OS are summarized in Table 3. Multivariable analysis showed that skeletal muscle loss of ≥3.24 cm^2^/month was independently associated with shorter OS (hazard ratio [HR] 5.74, 95% CI 2.94–11.24, *p* < 0.001), along with age ≥ 70 years (HR 1.95, *p* = 0.039), no response to first‐line therapy (HR 1.80, *p* = 0.044), and no second‐line therapy (HR 2.69, *p* = 0.001).

**TABLE 3:** Results of univariable and multivariable analyses of prognostic factor for overall survival

Variables	Univariable analysis		Multivariable analysis	
	HR (95% CI)	*p* value	HR (95% CI)	*p* value
Male	1.15 (0.54–2.43)	0.717		
Age ≥ 70 years	1.42 (0.87–2.30)	0.158	1.95 (1.04–3.68)	0.039
Stage IV	1.13 (0.54–2.36)	0.755	1.58 (0.71–3.49)	0.260
ECOG PS ≥2	1.46 (0.88–2.43)	0.143	0.92 (0.48–1.76)	0.796
Smoking (yes vs. no)	1.30 (0.62–2.75)	0.487		
Monotherapy vs. combination	1.33(0.69–2.55)	0.397		
Response to first‐line therapy (no vs. yes)	2.21 (1.31–3.72)	0.003	1.80 (1.02–3.17)	0.044
Receipt of second‐line therapy (no vs. yes)	3.16 (1.86–5.36)	<0.001	2.69 (1.48–4.89)	0.001
Sarcopenia	1.22 (0.65–2.28)	0.545		
Skeletal muscle loss ≥3.24 cm^2^/month (upper tertile)	3.84 (2.18–6.76)	<0.001	5.74 (2.94–11.24)	<0.001

*Abbreviations*: CI, confidence interval; ECOG PS, Eastern Cooperative Oncology Group performance status; HR, hazard ratio.

## DISCUSSION

We characterized cancer‐associated muscle wasting in patients with advanced SQCLC in this study. Patients with advanced SQCLC lose a significant amount of skeletal muscle until death (mean loss 16.5%). The rate of muscle depletion is accelerated at the end of life. These findings show that cancer‐associated muscle depletion progresses more rapidly in contrast to the physiologic age‐related muscle loss. A recent quantitative review reported that the rate of muscle loss throughout one's lifespan is 0.47% per year in men and 0.37% per year in women.^19^

Our study focused only on SQCLC among patients with NSCLC. Recently, it has been recognized that NSCLC is not a single disease entity.^20^ The subclassification of NSCLC has gained importance because of the therapeutic implications of the underlying histologic subtypes.^21^ Driver mutations applicable to molecularly targeted agents are usually detected in adenocarcinoma histology. The prognosis of patients with advanced SQCLC is poorer than that of patients with adenocarcinoma.^21^ A recent study showed that skeletal muscle loss during treatment was significantly different between patients receiving cytotoxic chemotherapy and tyrosine kinase inhibitors,^22^ with it being lower in patients receiving tyrosine kinase inhibitors (TKIs) than in patients receiving cytotoxic chemotherapy.

In our study, a higher amount of muscle loss after palliative chemotherapy was associated with a shorter survival in patients with metastatic SQCLC. This finding is in line with that reported for patients with different types of cancer, including foregut and colorectal cancers.^23^, ^24^, ^25^ In metastatic melanoma, patients with a loss of skeletal muscle ≥7.5%/100 days (the highest quartile) during immunotherapy showed a significantly increased risk of mortality (HR 2.1, 95% CI 1.02–4.55, *p* = 0.046).^26^ The worse prognosis in patients with higher muscle wasting may reflect aggressive tumor biology. Stene et al. suggested that there is an association between response to chemotherapy and changes in muscle mass in patients with disease control; 56% of patients with disease control following chemotherapy maintained skeletal muscle, whereas 80% with disease progression showed muscle loss.^2^ Furthermore, a recent study on patients with metastatic colorectal cancer indicated that the acceleration of skeletal muscle loss precedes disease progression.^27^

It is important to diagnose sarcopenia and evaluate the changes in skeletal muscle. However, the measurement is not routinely implemented at clinical practice because of limited time and human source to measure SMM. In this study, an artificial intelligence (AI) software based on deep learning technology was used for the automatic segmentation of skeletal muscle area and for diagnosing sarcopenia. CT is a useful tool for evaluating body components, and skeletal muscle area can be accurately quantified based on CT attenuation values (−29 to 150 HU for skeletal muscle). Semi‐automated segmentation methods using conventional software require additional manual correction of complex handcraft (10, 13–16). These approaches cannot be practically applied to longitudinal changes in large datasets. With the power of neural networks and convolutional layers to learn the hierarchy of features in a large amount of given data (17), a deep learning AI system can automatically and accurately segment and quantify skeletal muscle area using CT and can be adopted for clinical practice and various research purposes requiring body morphometry analysis.

The relationship between skeletal muscle loss and increased treatment toxicity, poor quality of life, and prognosis represents the need for early detection and therapeutic intervention of muscle wasting in cancer patients. Recent studies have shown that supervised exercise interventions are safe and feasible in advanced cancer patients.^28^ In addition, exercise interventions resulted in improvement in muscle strength, physical function, and quality of life.^29^ A randomized phase III trial of multimodal treatment consisting of nutrition, exercise programs, and anti‐inflammatory medication is underway.^30^ Concerning metastatic prostate cancer, a large phase III trial is ongoing to determine whether supervised high‐intensity aerobic and resistance exercise increase the OS.^31^

In summary, longitudinal skeletal muscle depletion determined using follow‐up serial chest CT progresses significantly until death in patienst with advanced SQCLC. Furthermore, our study suggests that a decrease in the extent of SMM and a high rate of SMM reduction could be used as a prognostic factor in managing SQCLC.

## DECLARATIONS

### Conflicts of Interest

The authors declare that they have no proprietary, commercial, or financial interests that could be construed to have inappropriately influenced this study.

### Ethics Approval

The institutional review board of GUGMC approved this retrospective study and waived the requirement for informed patient consent (approval number: GAIRB 2020‐447).

### Consent to Participate

As a retrospective study, the requirement for informed patient consent was waived.

## CONSENT FOR PUBLICATION

### Availability of Data and Material

Detailed patient data for participants in these studies is available and can be provided upon request.

### Code Availability

N/A

### Author Contributions

All authors participated in the concept/design, data interpretation, drafting, and critical revision of this article. The statistical analysis was conducted by Y. S. Kim.
